# The Stroke Outcomes Study 2 (SOS2): a prospective, analytic cohort study of depressive symptoms after stroke

**DOI:** 10.1186/1471-2261-9-22

**Published:** 2009-06-01

**Authors:** Kate M Hill, Robert M West, Jenny Hewison, Allan O House

**Affiliations:** 1 Leeds Institute of Health Sciences, The University of Leeds, Charles Thackrah Building, 101 Clarendon Road, Leeds, LS2 9LJ, UK

## Abstract

**Background:**

Mood disorder is recognised as an important and common problem after stroke but little is known about the longer term effects of mood on functional outcomes. This protocol paper describes the Stroke Outcomes Study 2 (SOS2), a research study conducted in two large acute NHS Trusts in the North of England, which was designed to investigate the impact of early depressive symptoms on outcomes after an acute stroke.

**Methods and design:**

SOS2 was a prospective cohort study that aimed to recruit patients in the first few weeks after a stroke, and to follow them up at regular intervals for one year thereafter in order to describe the trajectory of psychological symptoms and study their impact on physical functional recovery. Measures of mood and function were completed at baseline (approximately 3 weeks) and at four follow-up time-points: approximately 9, 13, 26 and 52 weeks after the index stroke.

**Discussion:**

Recruiting patients to research studies soon after an acute stroke is difficult. Mortality following stroke is approximately 30% and in the region of half the patients that survive the initial event are significantly disabled. Together these factors reduced the number of patients available to participate in SOS2 but once recruited to the study the drop-out rate was relatively low. During the recruitment period over 6000 admissions for stroke or query stroke were screened for eligibility. A cohort of 592 study participants was finally achieved.

## Background

Stroke is a complicated, heterogeneous condition with complex and enduring sequelae. It is increasing in incidence mainly as a consequence of the growing population of elderly people [1]. Improvements in acute stroke care have helped more people to survive the initial event but, while mortality has been reduced, stroke remains a major cause of disability. Half the survivors of the initial stroke event (about one third of all acute strokes) are left with some degree of physical disability, which can range in effect from moderate to severe. Some of the consequences of stroke while less apparent are nonetheless important; mild cognitive impairment for example can make some of the tasks of daily living difficult, and loss of role and physical function can have profound psychological effects on stroke survivors.

Depression is an important contribution to poor quality of life. It occurs in about a third of stroke survivors in the first months after stroke [2] and antidepressant medication is widely used in clinical practice although the evidence that it is effective is surprisingly weak. [3] Less is known about the longer term effects of depression, or its treatment, on functional outcomes. This study (SOS2) was undertaken because few studies have explored the association between depressive symptoms as they evolve over time after stroke and later functional outcome. The study was designed to investigate the impact of emotional disorder on recovery from stroke prospectively by identifying depressive symptoms in the early weeks after stroke and tracking patients' mood and functional status over a one year period. A randomised placebo- controlled trial of an SSRI anti-depressant was nested in the cohort study. This study has been reported in full elsewhere [4].

## Methods

### Setting

The study was conducted in three Acute and four Rehabilitation Units in two NHS Trusts in the West Yorkshire area. A hospital admission related to the stroke event was therefore one of the inclusion criteria and participants were not drawn from the estimated 500 to 600 patients per year who are treated by generic services in the community. In practice however many of the follow-up interviews and some initial interviews were actually conducted in the community, usually in the patient's own home. The study had full approval from the relevant Local Research Ethics Committees (LREC) in the areas in which it was conducted. Project reference numbers:

Leeds Teaching Hospitals NHS Trust:

St. James's University Hospital LREC      Ref No:   01/182

Leeds General Infirmary LREC         Ref No:   CA02/131

Bradford Hospitals NHS Trust LREC      Ref No:   02/06/222

### Sample

Patients were recruited prospectively following an admission to either of the two acute trusts with new or recurrent stroke between 1 July 2002 and 31 March 2005. Inclusion criteria were as follow:

• Life-time first or recurrent stroke survivor and fit to be seen at 2 – 4 weeks;

• Adult aged 18 years or older;

• Able to give informed consent;

• Post-consent: an MMSE (Mini Mental State Examination) [5] score of 23 or above (Borderline scores between 20 and 23 were accepted if physical or speech deficits contributed to low scores).

Exclusion criteria were:

• Subarachnoid haemorrhage or transient ischaemic attack;

• Severe cognitive impairment;

• Concurrent major illness the management of which was likely to predominately determine care;

• Non-English speaking.

Systematic screening of the admissions records on numerous wards was required to find patients within the relatively short timeframe available from onset of stroke (2 to 4 weeks). Patients were tracked through the hospital ward network and beyond, to home or residential care homes, if they had already been discharged. The medical reception units provided an initial starting point from which to trace potential participants as most emergency cases are admitted via these wards before being dispersed to other locations. Routine trawling of all other wards where stroke patients could be admitted, for example the elderly, general medical wards and the specialist stroke units, was also performed to ensure that direct admissions from A&E or by GP referral were not missed. Information on patient movement was obtained from admission and discharge registers on the wards, and from the nursing or medical staff.

By carrying out a broad sweep of hospital admissions we aimed to ensure that stroke patients who had spent only a short time on a general medical ward or those who were admitted for longer periods of time to non-specialised, particularly elderly care, wards were not missed. This approach enabled a sample to be accrued that was representative of all types of stroke patient presenting at hospital, and not just those who were admitted directly to Acute Stroke Units or specialised Stroke Rehabilitation Units.

### Study Design

The nature of the investigation required that patients be recruited within two to four weeks after the index stroke event in order to identify early onset of depressive symptoms. Once first stage eligibility had been determined the patient was contacted by a member of the research team. If the patient remained in hospital initial contact was made by introduction from a member of the care team. If they had been discharged home they were contacted directly either by letter or telephone. All patients received a written information sheet and had the study fully explained to them by a researcher. They were also given an opportunity to ask questions about the study before being asked to sign a consent form. The MMSE was then applied before proceeding with the full baseline assessment. Some flexibility in the timing of the baseline interview (up to 6 weeks post-stroke) and the cut-off point for the MMSE was permitted in order to include a range of stroke severities, and to meet recruitment targets.

Baseline interviews (T1: 2 to 6 weeks) were conducted with consenting patients either in hospital, in care homes or their own residences. Follow-up occurred at 4 additional time points: T2 (8 to 10 weeks); T3 (12 to 14 weeks); T4 (24 to 28 weeks) and T5 (52 weeks) after stroke. One patient preferred to attend the research office to complete the follow-up assessments.

### Study Measures

After consent was obtained, a comprehensive interview was completed at T1 to collect information on socio-demographic, physical functioning and psycho-social measures. Relevant clinical information and details of any comorbidity were obtained from medical records also with the consent of the patient. Psychological well being was assessed at each visit using the self-reported 28 item General Health Questionnaire (GHQ28) [6] and the Present State Examination [7].

Four domains, each containing seven items, are represented in the GHQ_28: somatic symptoms, anxiety, social dysfunction and depressive symptoms. For the purpose of this study the items were rated by the patient on a four point scale and scored in two ways: either bimodally (0-0-1-1) for symptom present or absent or categorically from 0 (not at all) to 3 (much more than usual).

The PSE is an interview schedule which was delivered to the patient as a series of verbal questions and probes. The responses were rated by the interviewer on a scale 0 to 2 to indicate the presence and severity of psychological symptoms on the basis of the information provided by the patient.

Physical functioning was measured by the Barthel Index [8] at each time point in the study. Other measures of functional and physical status, health related quality of life and motivation were applied at various time-points. These are listed below:

The Glasgow Coma Scale [9]

WHO Performance Status [10]

Frenchay Aphasia Screening Test [11]

Rivermead Mobility Index [12]

Duke's Severity of Illness Scale [13]

Frenchay Activities Index [14]

Functional Independence Measure [15]

A therapist rated measure of participation in rehabilitation [16]

The MOS Short-Form 36-item Questionnaire [17]

Table 1 shows the time points in the study and the measures used at each stage.

**Table 1:** Timetable of assessments in the SOS2 study

**BASELINE ASSESSMENT (T1)**	**6 WEEKS (T2)**	**3, 6 AND 12 MONTHS** **(T3, T4 AND T5)**
Demographic data: age, sex, residential status, occupation etc. Current mediation. Smoking history.	Current mediation Place of residence	Current mediation Place of residence
		
***Impairment***	***Impairment***	***Impairment***
		
Glasgow coma scale on admission] Urinary continence in the first 2 weeks Presence of hemianopia WHO Performance Status ratings	WHO Performance Status rating	WHO Performance Status rating
		
***Cognition***		
		
Mini Mental State Examination (MMSE) – a brief screening for cognitive impairment Frenchay Aphasia Screening Test	*If cognitive state changed or in doubt * repeat Mini Mental State Examination (MMSE)	*If cognitive state changed or in doubt * repeat Mini Mental State Examination (MMSE)
		
***Functioning***	***Functioning***	***Functioning***
		
The Barthel Index – pre- and post-stroke Rivermead Mobility Index	Barthel Index Frenchay Activities Index (FAI) – if the patient is at home Functional Independence Measure (FIM) Therapist-rated measure of participation in rehabilitation (If applicable)	Barthel Index Frenchay Activities Index (FAI) – if the patient is at home Functional Independence Measure (FIM) Therapist-rated measure of participation in rehabilitation (If applicable)
		
***Mood***	***Mood***	***Mood***
		
GHQ-28 – a self report mood rating scale Modified short form Present State Examination (PSE) – a standardised psychiatric interview to derive ICD-10 diagnoses (with additional questions on alcohol consumption and emotionalism)	GHQ-28; Modified short-form PSE	GHQ-28; Modified short-form PSE
		**Quality of Life**
		SF-36: a self-reported measure of health-related quality of life
		
***Co-morbidity***		
		
Dukes Severity of Illness Scale		

### Recruitment rates

The pattern of recruitment at ward level showed that while the majority of patients (68%) were recruited from the specialist stroke wards, a miscellany of wards collectively contributed a significant number of recruits (32%). Several explanations can be offered for this pattern: firstly at times when bed occupancy was high in the acute hospitals, stroke patients requiring hospital admission could be sent wherever a bed was available; secondly mild stroke sufferers admitted for observation or assessment were not transferred to stroke units because they did not require their specialised services; thirdly and conversely, severe strokes with major physical impairments were not always admitted to stroke wards if nursing staff resources were already at their limit for managing the levels of dependency on the ward. Patients in the final category were usually transferred to the Stroke Unit as soon as it was feasible to do so.

**Figure 1:**
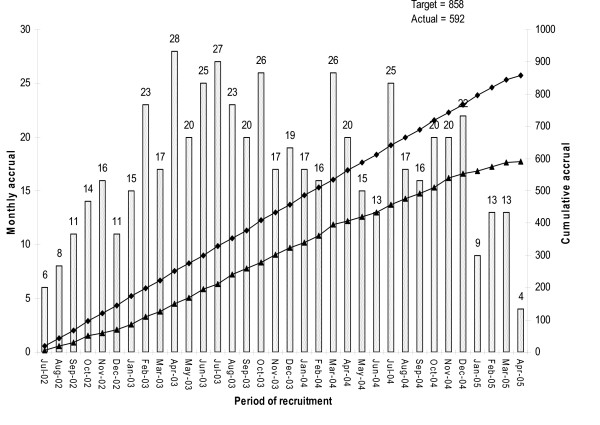
**Final recruitment graph showing monthly and cumulative accrual**.

The target for the study was to accumulate a sample of approximately 900 patients into the main cohort during the three year period of active recruitment. Figure 1 shows the actual and predicted monthly accrual during the active phase of the study.

### Exclusions

Table 2 lists the exclusion categories and gives a breakdown of the number of patients in each of them. Post-stroke mortality and impaired cognition were the main clinical categories under which patients were excluded. All eligible patients were invited to participate in the study as they presented and asked for written consent if they agreed. Refusal to enter the study accounted for a loss of almost half (45%) of the eligible patients. Table 3 shows the reasons given by patients for refusing consent.

**Table 2:** Exclusion Categories

	**N**	**%**
**Clinical factors**		

Subarachnoid Haemorrhage	51	2.5
Deceased	425	20.9
Co-morbidity	145	7.1
Too poorly or too frail	218	10.7
Dysphasia/Aphasia	206	10.2
Deafness	14	0.7
Impaired cognition	431	21.1

**Administrative factors**		

Too young	1	0.05
Non-English speaking	128	6.3
Out of area	41	2
Unable to contact	92	4.5
Admission > 4 wks post stroke or unconfirmed diagnosis within 4 wks	204	10
Missed – holidays/absences	77	3.8
SOS2 already completed	4	0.2
Previously withdrawn	1	0.05

**Total number of patients excluded**	2038	100

**Table 3:** Refusal Categories

	**N**	**%**
No reason given	163	34.0
Not interested	108	22.6
Feels too unwell/unable to cope	123	25.7
Feels limited by speech impairment	23	4.8
Too busy with other commitments	31	6.5
Feels too old	25	5.2
Denies any stroke	5	1.0

**Total refusals**	478	100%

**Figure 2:**
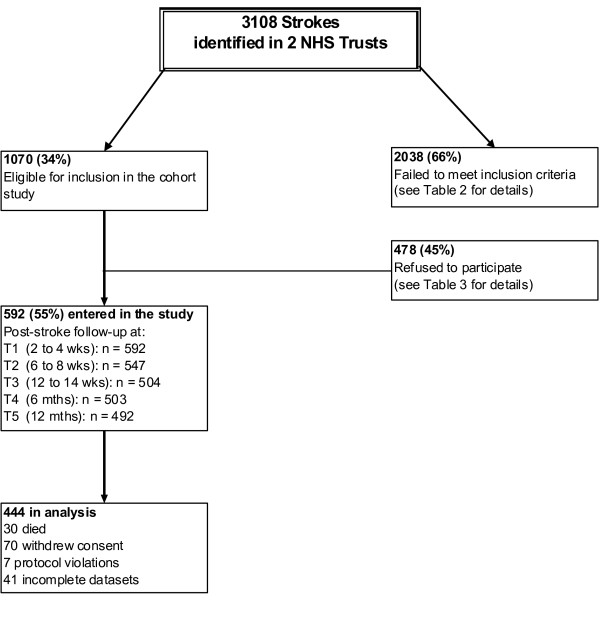
**Flow diagram of recruitment to the main cohort (SOS 2)**.

A cohort of 592 patients was finally achieved which was less than the predicted sample size. The shortfall in the total number of people recruited to the study can be attributed to a number of factors but was due in part to higher than predicted levels of ineligibility and the relatively high refusal rate among the target population. Recruiting soon after stroke also affected participation in the study, and staff resources within the research team also limited recruitment at certain times. Figure 2 shows a flow diagram of the recruitment process.

### Data quality

The research team were subject to induction training in study procedures and the conduct of the research interview including a period of specific training in the use of the Present State Examination. Standardisation across the team was maintained through weekly team briefing meetings. Data were collected on a series of structured assessment sheets specifically designed for the study. Interviewers were responsible for checking that self-completed assessment sheets were correctly filled in before finishing the interview visit with the patient.

A full data check was carried out during the final year of the study. All data sheets were examined for completeness for each of the participants and a sample of 10% of the data re-entered onto the database to check for accuracy. Missing values were entered if the data were present or could be found. The double entry procedure detected an error rate of 9.6% in the sample tested. Eighty per cent of errors found were associated with the PSE, which required scores to be transposed from the 32-page schedule to a score sheet for data entry. After the full sample had been checked, all PSE schedules for the remaining patients were checked against the score sheets and any errors corrected on the database.

### Statistical analysis

Repeated measures enabled the assessment of mood throughout rehabilitation. The original plan of analysis was to conduct multi-level modelling of the data to examine the effect of early depressive symptoms on later outcomes. The analytical strategy was revised as the study progressed and a decision made to broaden the scope of the analysis in order to explore symptom trajectories and their effects on outcomes. The objective of the analysis was to identify clusters of patients representing patterns of mood symptoms during rehabilitation [18-20]. This approach had the benefit of yielding latent classes that could be used in a regression model in place of repeated measures of mood thus avoiding the problems of co-linearity that would have arisen with highly correlated variables. The clusters themselves thus became useful secondary outcomes of the analysis. A further benefit of the latent-class approach was that greater power could be delivered. Effectively there are *m*-1 covariates to be fitted to final logistic regression where *m *is the number of latent classes. In practice *m *will not be taken to be too large or otherwise the classes become less valuable.

The dichotomous outcome was taken as attaining a Barthel score of 20 (best) or not. Thus the power of the study depended upon the ratio of the number of events (Barthel = 20), or non-events if this is smaller, to the number of classes *m*. In practice there were 261 events and *m *= 5 classes were used. This provided a ratio of 261/4 = 65, in excess of the value 10 recommended by Peduzzi *et al*. (1996). [21]

## Discussion

### Sample

Early observations on stroke patients' ward transitions from admission through A&E showed that if recruitment was confined to patients from designated stroke units alone, a large proportion of patients admitted with stroke (or query stroke) would be missed. It is likely too that a sample with a different profile would have resulted, as the more severe strokes were usually given priority for stroke unit beds (when available). Our policy of wider recruitment thus ensured access to a greater number of potential recruits, representative of the stroke population typically passing through an acute hospital.

Overall the cohort recruited to our study was relatively less disabled than the total population of stroke survivors. Our recruitment policy may have given more minor stroke patients the opportunity to participate but it is also probable that the design of the study played a role in determining the profile of our cohort. We wanted to study depressive symptoms soon after stroke and consent therefore had to be obtained (and the baseline interview had to be conducted) in the early weeks following the index stroke. This method effectively excluded some of the more severe strokes, and the older and frailer patients who were not willing or able to participate in the early weeks after their stroke event.

Furthermore the latent class modelling required that the subjects included in the analysis were those for whom assessments at all five time points were available. This also had the effect of selecting the fitter and more able cases as these were the least likely to have missed visits during the study.

Ethnic minorities were significantly under-represented despite specific efforts made to recruit from these groups. Only one patient of Asian origin and three Afro-Caribbean patients were entered into the study; unfortunately one of these patients died and two withdrew before the one year follow-up point.

### Significance of the study

There is existing evidence to demonstrate that mood disorder is commonly associated with stroke [2], which is not surprising given the sudden and acute onset of the stroke event, and the consequent impact that it can have on day to day activities and quality of life. Less is known about the pattern of depressive symptoms after a stroke and the way in which they evolve. The study described here was designed to investigate the nature of mood disorder associated with stroke more closely and to examine its effect on the rehabilitation potential of patients over the course of a year. A particular feature of this study was the longitudinal design, an approach that enabled latent class analysis to be applied. This is a relatively novel way of characterising mood disorder, which is more often treated as a single, event-like exposure.

## Competing interests

The authors declare that they have no competing interests.

## Authors' contributions

AH had the original idea for the study, and contributed to design, implementation and interpretation, and preparation of the manuscript. JH contributed to the design and interpretation of the study. AH and JH obtained the funding necessary to conduct it. RW redesigned the analytical approach and undertook the statistical modelling. He also contributed to writing the paper. KH operationalised and coordinated the study, supervised the research team, and managed the data. She undertook the preliminary data analyses, contributed to the interpretation of the data and led on writing the paper. All authors read and approved the final manuscript.

## Pre-publication history

The pre-publication history for this paper can be accessed here:


